# Biomarkers for Heart Failure Prognosis: Proteins, Genetic Scores and Non-coding RNAs

**DOI:** 10.3389/fcvm.2020.601364

**Published:** 2020-11-23

**Authors:** Apurva Shrivastava, Tina Haase, Tanja Zeller, Christian Schulte

**Affiliations:** ^1^Clinic for Cardiology, University Heart and Vascular Center, University Medical Center Eppendorf, Hamburg, Germany; ^2^German Center for Cardiovascular Research (DZHK), Partner Site Hamburg/Kiel/Luebeck, University Medical Center Eppendorf, Hamburg, Germany; ^3^King's British Heart Foundation Centre, King's College London, London, United Kingdom

**Keywords:** biomarker, heart failure, prognosis, protein biomarker, NT-proBNP, non-coding RNA, genetic risk score

## Abstract

Heart failure (HF) is a complex disease in which cardiomyocyte injury leads to a cascade of inflammatory and fibrosis pathway activation, thereby causing decrease in cardiac function. As a result, several biomolecules are released which can be identified easily in circulating body fluids. The complex biological processes involved in the development and worsening of HF require an early treatment strategy to stop deterioration of cardiac function. Circulating biomarkers provide not only an ideal platform to detect subclinical changes, their clinical application also offers the opportunity to monitor disease treatment. Many of these biomarkers can be quantified with high sensitivity; allowing their clinical application to be evaluated beyond diagnostic purposes as potential tools for HF prognosis. Though the field of biomarkers is dominated by protein molecules, non-coding RNAs (microRNAs, long non-coding RNAs, and circular RNAs) are novel and promising biomarker candidates that encompass several ideal characteristics required in the biomarker field. The application of genetic biomarkers as genetic risk scores in disease prognosis, albeit in its infancy, holds promise to improve disease risk estimation. Despite the multitude of biomarkers that have been available and identified, the majority of novel biomarker candidates are not cardiac-specific, and instead may simply be a readout of systemic inflammation or other pathological processes. Thus, the true value of novel biomarker candidates in HF prognostication remains unclear. In this article, we discuss the current state of application of protein, genetic as well as non-coding RNA biomarkers in HF risk prognosis.

## Introduction

Heart failure (HF) is a complex cardiovascular disease (CVD) in which the heart's functional capacity is reduced, leading to failure in meeting the body's blood and oxygen demand (1). The most common risk factors are age, sex, environmental risk factors, genetic disposition, and diseases such as diabetes, hypertension, coronary artery disease, and atrial fibrillation. HF is described as a global pandemic as it affects ~26 million people worldwide (2). In North America and Europe, >80% of HF cases comprise of people who are ≥65 years old (2–5). Survival rate of HF patients is poor with 2–17% of HF patients dying while in hospital, 17–45% patients die within 1-year of admission and the majority dies within 5-years of admission (6). Due to the high mortality rates associated with HF, early diagnosis of the first subclinical signs is essential to prevent severe outcomes.

HF is a multi-system disorder which is characterized by a decrease in functional capacity of the heart. Reduced cardiac output due to impairment in left ventricular function leads to an activation of the neuro-hormonal system. This, in turn, stimulates the renin-angiotensin-aldosterone system leading to increased concentrations of renin, angiotensin II and aldosterone, each of which causes salt and water retention, vasoconstriction, and enhanced sympathetic activity. Prolonged exposure to neuro-hormonal activation leads to dilation and structural changes in the myocardium and fibrosis, thereby further worsening cardiac output (7). The severity of HF is graded in accordance to the New York Heart Association (NYHA) classification I, II, III, and IV. This gradation is based on patient clinical symptoms and effect of HF on their physical mobility. This definition also takes into account the decrease in the left ventricular ejection fraction (LVEF), classifying HF as either HF with preserved ejection fraction (HFpEF; LVEF ≥50%) or HF with reduced ejection fraction (HFrEF, LVEF <50%). HF involves micro- and macro-structural changes, each involving activation of inflammatory and neuro-hormonal system that release several biomolecules to compensate for the failing heart. Consequently, a storm of cytokines and regulatory molecules are released. The abundance of dysregulated molecules make it difficult to identify biomarkers that can specifically aid in HF prognosis.

**Figure 1:**
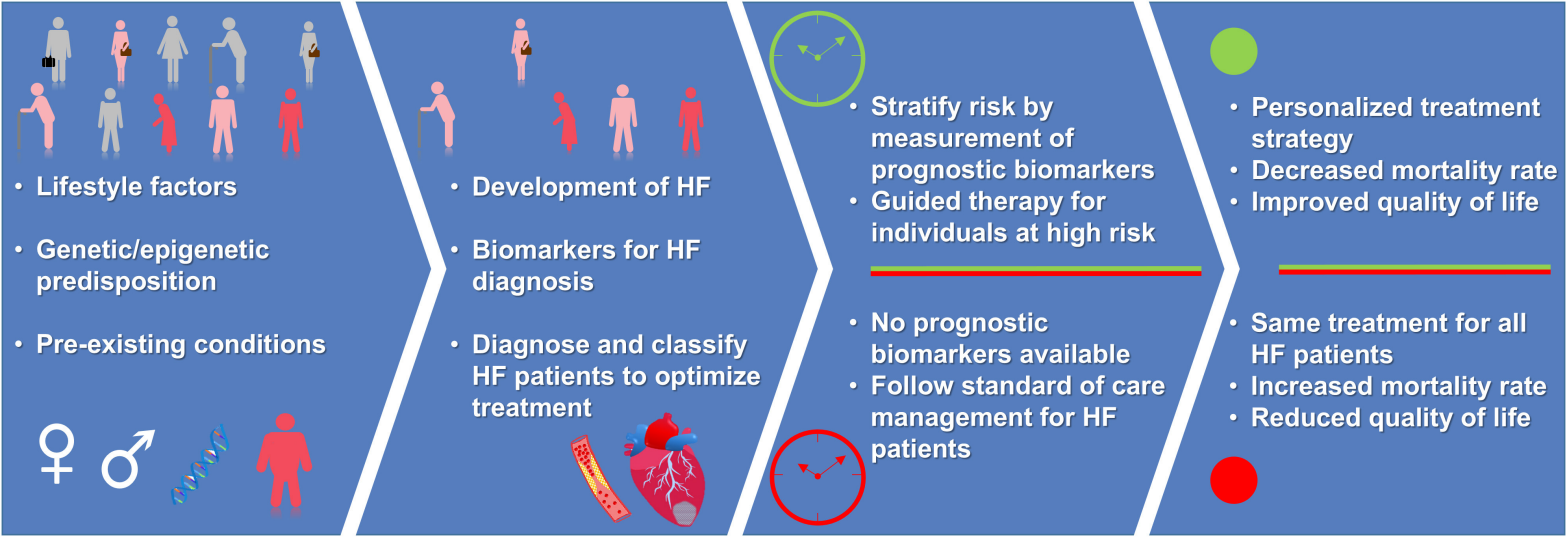
Advantage of having a prognostic biomarker for personalized treatment strategy. Presence of prognosis biomarkers allows for early identification of individuals at-risk of developing HF. For individuals with HF, measurement of prognosis biomarkers guide the treatment strategy leading to reduced risk of mortality and improved quality of life (green color), when compared to absence of prognosis biomarkers and standard of care management (red color). Depiction of human figures: pink, humans at-risk of HF; red, humans having HF; gray, healthy humans.

A biomarker is defined as a biological compound that is easily accessible and measurable in the body. The biomarker can be classified as molecular, cellular, or imaging as long as they help in identifying the disease or provide therapeutic guidance. Diagnosis of HF through different diagnostic tools such as chest X-ray (8, 9), echocardiography (ECG) (10–12), and cardiac magnetic resonance (CMR) (13–15), have been highly reliable in guiding treatment (16). Imaging biomarkers provide great insight into the structural and functional abnormalities of the heart, however, the imaging biomarker readout fails to detect early and subclinical states of HF. Moreover, the reliability of imaging biomarker readout is biased depending on image quality, imaging modality, the observer, and center experience, as there exist differences based on age, sex, and imaging modality (16). Natriuretic Peptides (NP), i.e., brain-type natriuretic peptide (BNP) and N-terminal prohormone of BNP, and cardiac troponin measurements have been included in the guidelines for HF diagnosis and treatment of the European Society of Cardiology (ESC) (16) and the American Heart Association (AHA) (17). Addition of other diagnostic biomarkers such as markers of inflammation (e.g., soluble ST2 receptor), oxidative stress (e.g., growth differentiation factor-15) and cardiac remodeling (e.g., galectin-3) can be beneficial in guiding HF therapy (17). Though there are several well-established diagnostic biomarkers for HF, the prognostic biomarkers for such a complex disease still remain poorly evolved. Therefore, in addition to existing imaging diagnosis techniques, it is important to identify biological markers which focus on HF pathogenesis and molecular function that can aid in risk stratification and patient care (Figure 1).

In this review article, we elucidate the role of the most prominent circulating protein biomarkers which show promising results in HF prognosis. In addition, we provide details on genetic biomarkers and polygenic risk scores that are currently being developed along with details about emerging evidence on circulating non-coding RNA biomarkers.

## Protein-Based Biomarkers

**Figure 2:**
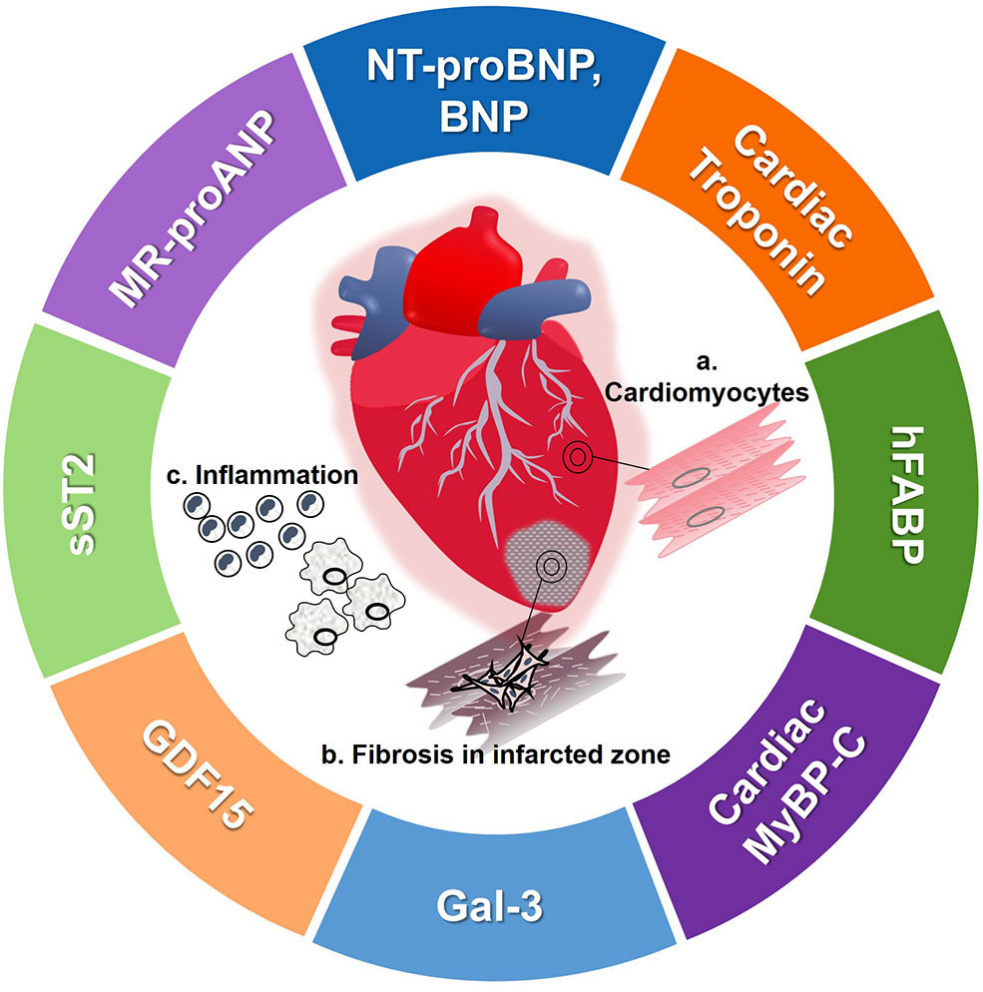
Pictorial representation of protein biomarkers detailed in this paper. **(a)** During HF, cardiomyocyte damage and restructuring leads to active and passive release of cardiomyocyte specific biomarkers. **(b)** After an event of myocardial infarction, the cardiomyocytes are destroyed and fibrosis occurs. **(c)** HF activates inflammatory pathways which also release other biomarkers, representative of systemic inflammation. BNP, brain-type natriuretic peptide; Gal-3, galectin 3; GDF15, growth differentiation factor 15; hFABP, heart-type fatty acid binding protein; MR-proANP, mid-region of N-terminal prohormone of atrial-type natriuretic peptide; MyBP-C, myosin binding protein-C; NT-proBNP, N-terminal prohormone of BNP; sST2, soluble suppression of tumorigenicity 2.

Protein biomarkers are released into the circulation and can be detected using various assays. Protein biomarkers that have entered the prognostic field for HF are either released from the heart exhibiting its value of tissue-specific damage, or are released from other cells as a systemic response to HF (Figure 2). In addition to tissue specificity, the half-life of protein biomarkers is often the crucial factor for its potential use as a biomarker (Table 1). The ease of measurement of circulating protein biomarkers and the speed of assay results make them invaluable to HF diagnosis and prognosis.

**Table 1:** Basic information on the prognostic/diagnostic capability, organ/cell of origin of protein biomarkers that are currently used for HF prognosis or diagnosis.

**Biomarker**	**Prognostic/diagnostic**	**Organ/cell of origin**	**Mode of release**	**Half-life**
NT-proBNP	Prognostic and diagnostic	Cardiac ventricles	Actively upon cardiomyocyte injury	120 min
MR-proANP	Prognostic and diagnostic	Cardiac atrial	Actively upon cardiomyocyte injury	60–120 min
Troponin I, T	Diagnostic and maybe prognostic	Cardiac thin filament	Passively upon myofibrillar injury	120 min
cMyBP-C	Diagnostic	Cardiac thick filament	Passively upon myofibrillar injury	Not known
hFABP	Prognostic	Cardiomyocyte cytoplasmic protein	Passively upon cardiomyocyte membrane injury	27 min
Gal-3	Diagnostic	Multiple cells	Upon inflammation and cardiac remodeling	Not known
GDF-15	Diagnostic	Multiple cells	Not clear	Not known
sST2	Diagnostic	Cardiomyocytes, cardiac fibroblasts, and vascular endothelial cells	Upon fibrosis and cardiac remodeling	Not known

### Natriuretic Peptides

Natriuretic peptides (NPs) are hormones that play an important role in regulating volume and pressure overload, thereby maintaining homeostasis within the cardiovascular system (18). NPs are initially produced as pre-prohormones which are then cleaved to form prohormones and later the final hormones. Atrial NP (ANP) released from cardiac atria, brain NP (BNP) released from cardiac ventricles and C-type NP (CNP) are the three different types of NP hormones. This NP system includes three NP receptors (NPRs) namely NPR-A (or guanylyl cyclase A), NPR-B (or guanylyl cyclase B), and NPR-C (or clearance receptor) (19). Both ANP and BNP have similar physiological roles; they act by binding to NPR-A (19) inducing diuresis, natriuresis, and vasodilation in an attempt to reduce cardiac preload. Furthermore, they inhibit aldosterone synthesis and renin secretion, which reduces cardiac afterload (19–21). ANP, BNP and CNP bind to NPR-C which plays a role in their clearing from the system by receptor internalization and hormone degradation (18, 19).

#### BNP and N-Terminal Prohormone of BNP

BNP, encoded by the *NPPB* gene, is secreted in response to increased ventricular stretching (22). BNP acts by binding to NPR-A on target tissues, activating the cGMP signaling cascade to reduce vascular stress, diuresis, natriuresis, and inhibition of renin-angiotensin-aldosterone system (23, 24). BNPs are present in a healthy individual at ~3.5 pg/mL in plasma, which increases by 100-fold in HF patients (25). However, BNP has a short half-life of 20 min when compared to the prohormone form of BNP known as N-terminal prohormone of BNP (NT-proBNP) with a longer circulation half-life of 60–120 min (26) (Table 1). Therefore, NT-proBNP is widely used in clinical assessment of HF.

There are numerous studies that have evaluated the prognostic ability of NT-proBNP and BNP biomarkers in various HF settings such as acute or chronic HF, providing robust evidence of their incremental value (27–30). BNP was measured in a study consisting of 122 acute decompensated HF patients along with worsening renal function. A significant reduction in BNP value of ≥40% over the period of hospitalization i.e., from baseline to discharge had a positive prognostic value in reduced rehospitalization (31). The PARADIGM-HF trial (2016) quantified NT-proBNP levels in 2,080 HF patients. Patients with reduction in NT-proBNP levels had a subsequent lower rate of HF hospitalization and CV death (32). Later in 2019, the PARADIGM-HF trial tested the prognostic value of BNP and NT-proBNP before and during treatment with neprilysin inhibitors (sacubitril/valsartan) in HF. From a patho-mechanistic point of view, the authors reported that the treatment caused a direct inhibition of neprilysin leading to a treatment-associated increase in circulating BNP levels early after treatment initiation. This report indicates that inhibiting neprilysin causes a direct increase of BNP in the circulation, while NT-proBNP was not affected. Therefore, the group concluded that the use of NT-proBNP as a biomarker (which is not a substrate for neprilysin inhibition) rather than BNP avoids clinical confusion (33). The combination of biological pathways and the reported clinical findings strengthen NT-proBNP's position as an excellent circulating biomarker for HF. Additionally, serial measurements of NT-proBNP at the time of hospital presentation and over the course of treatment has the potential to provide better prognostic information on disease outcome. In the TRANSITION study (2020), NT-proBNP was studied at the time of randomization, after 4 weeks and after 10 weeks in 982 HFrEF patients with acute decompensated HF. Reduced NT-proBNP levels at 4 weeks was indicative of a lower risk of HF re-hospitalization and CV death (hazard ratio: 0.57; 95% confidence interval (CI): 0.38–0.86; *p* = 0.007). In another prospective multi-center study of 171 patients with acute decompensated HF, BNP, and NT-proBNP measurements were taken at the time of hospital presentation, after 24 h, after 48 h and at the time of discharge. The primary end point (1-year all-cause mortality) was reliably predicted by BNP and NT-proBNP, with the prognostic accuracy of both biomarkers increasing over the course of hospitalization. The area under the receiver operating curves (ROC) curve (AUC) increased during the course of hospitalization for BNP (AUC presentation: 0.67; AUC 24 h: 0.77; AUC 48 h: 0.78; AUC discharge: 0.78) and NT-proBNP (AUC presentation: 0.67; AUC 24 h: 0.73; AUC 48 h: 0.75; AUC discharge: 0.77) (34). This observation seems comprehensible given inter-individual differences in the severity of the disease upon presentation to hospital, with these differences being reduced upon HF treatment. It is to be noted that the reported AUC values with a maximum of <80% don't seem to support BNP or NT-proBNP as clinically applicable biomarkers—at least not when applied as single markers.

NT-proBNP has further been evaluated as a means of guiding HF therapy in various studies with mixed results: The PROTECT study (2014) investigated whether lowering of NT-proBNP concentration (NT-proBNP guided therapy) led to an improvement in echocardiographic parameters when compared to standard of care management in 151 chronic HF patients with LV systolic dysfunction. The authors reported that guiding therapy through NT-proBNP levels demonstrated prognostically meaningful improvement in echocardiographic parameters such as LV volumes and function (35). By contrast, when treatment of *n* = 405 acute decompensated HF patients was guided by NT-proBNP, 6-month outcome was not improved in the PRIMA II trial (2018) (36).

BNP and especially NT-proBNP are reliable gold standard diagnostic biomarkers in heart failure, likely driven by their well-understood and cardiac-specific biological function. Their prognostic potential seems promising, while at the same time, when used as single markers, their accuracy doesn't yet allow for clinical applicability and their use in HF treatment guidance still needs to be further evaluated.

#### Mid-Region of N-Terminal Prohormone ANP (MR-proANP)

ANP, encoded by the *NPPA* gene, is secreted in response to atrial volume stretch receptors (37). The physiologic activity of ANP is similar to BNP, ANP acts by binding to NPR-A in cardiac atria, kidney, adrenal glands, and vascular smooth muscle cells, causing an increase in renal sodium excretion. This results in decreased extracellular fluid volume and blood volume, thereby improving cardiac ejection fraction and reduction of blood pressure (38). In healthy individuals, plasma levels of ANP are ~20 pg/mL and it rises 10–100 times in HF patients (39). It is difficult to measure the bioactive form of ANP due to its short half-life (2 min) (40–43) and although the N-terminal prohormone form of ANP (NT-proANP) is more chemically stable, it is easily degraded (40). Therefore, mid region of NT-proANP (MR-proANP) which is less susceptible to proteolytic degradation is used in clinical assessment (40, 44).

MR-proANP retains its prognostic incremental value in regards to the study end-points and has mostly been studied in combination with NT-proBNP and/or other biomarkers. In acute HF patients, higher MR-proANP levels provided long-term (5-year follow-up) prognostic value [MR-proANP (AUC = 0.668) vs. BNP (AUC = 0.604), *p* = 0.042 and vs. NT-proBNP (AUC = 0.564), *p* = 0.004] (45). A secondary analysis from the MOLITOR trial (2019) investigated serial measurements of MR-proANP and NT-proBNP biomarkers in 104 acute decompensated HF patients for betterment in quality of life (QoL). The authors concluded that MR-proANP predicted the reduced physical and mental QoL whereas NT-proBNP was predictive of reduced physical QoL (46). In the GISSI-HF trial (2010) investigators quantified MR-proANP along with three other circulating biomarkers in 1,237 chronic and stable HF patients at baseline and 3-months follow-up. Serial measurements of MR-proANP had the best prognostic accuracy with AUC of 0.74 (95% CI = 0.71–0.77) independent of NT-proBNP, which had an AUC 0.73 (95% CI = 0.70–0.76) (47). Similar results were reported in other clinical trials (48, 49). Thus, while not as clinically established as NT-proBNP in the diagnostic field, there is some evidence that MR-proANP may serve as an equally precise prognostic biomarker in HF patients.

NPs function as cardiac-specific hormones, released in pathologic states of increased cardiac strain. Therefore, their biomarker function is directly derived from a cardiac-specific pathology and comprises a strong link to a natural therapeutic attempt. This may well be an important reason why NPs remain unparalleled in the field of biomarkers for heart failure diagnosis, prognosis, and now also in guided therapy (18).

### Troponin I and T

Troponin protein is a component of skeletal and cardiac muscle thin filaments and has three isoforms, namely troponin C (Tn-C), troponin I (Tn-I encoded by *TNNI3* gene) and troponin T (Tn-T encoded by *TNNT2* gene), known as the troponin complex. Cardiac isoforms of Tn-I (cTn-I) and Tn-T (cTn-T) are exclusively expressed in cardiac muscle, whereas Tn-C is expressed as one isoform in both cardiac (encoded by *TNNC1* gene) and skeletal muscle (encoded by *TNNC2* gene). The troponin complex plays a key role in cardiac and skeletal muscular contraction (50), regulated by calcium concentration in the myocyte cytoplasm. This is achieved by allowing calcium binding (by Tn-C), inhibition of ATPase activity of actomyosin complex (by Tn-I) and interaction with tropomyosin which is wrapped around the actin (by Tn-T). cTns are the primary biomarker for MI and acute coronary syndrome (ACS) and can also be elevated during HF. Therefore, cTns present a picture of myocardial damage and not HF itself, and are known as cardiac injury biomarkers. Guidelines recommend measuring cTn along with NPs at the time of hospitalization to identify acute coronary syndrome as the underlying cause of acute HF patients as well as for prognosis of HF disease (16, 17).

The ASCEND-HF study examined the prognostic importance of cardiac troponin I (cTn-I) in patients with acute decompensated HF. Elevated levels of cTn-I (above 99% upper reference limit) was observed in 50% of acute decompensated HF patients and helped in predicting in-hospital outcome (p=0.01), but was not an independent predictor of post discharge outcomes (51). The CORONA trial examined high-sensitivity cardiac troponin T (hs-cTn-T) in a subgroup of elderly patients (≥60 years, *n* = 1,245) with systolic HF of ischemic origin. The authors reported that elevated hs-cTn-T levels (above 99% upper reference limit) provided a strong and independent prognosis of CV death, non-fatal MI, non-fatal stroke and HF hospitalization in older patients with chronic ischemic HF (52). The RELAX-AHF study investigated the association between serial measurements (baseline, 2, 5, and 14 days) of hs-cTn-T and outcomes [CV death (180 days), HF/renal hospitalization (60 days), and dyspnea relief]. The authors concluded that hs-cTn-T was elevated above 99% upper reference limit in 90% of patients. Higher levels of hs-cTn-T was associated with worse outcomes and CV death (180 days) (hazard ratio adjusted hs-cTnT = 1.36, 95% CI 1.15–1.60, *p* = 0.0004) (53).

When measured with other biomarkers, Tn-T retained its prognostic value. The RED-HF trial tested the prognostic values of various cardiac inflammatory and renal biomarkers in HFrEF patients. The authors concluded that NT-proBNP and hs-Tn improved risk stratification in HFrEF subjects, outperforming other emerging biomarkers tested by the group (54). A biomarker sub-study of the PARADIGM-HF trial investigated the prognostic importance of NT-proBNP and Tn-T measurements in HF patients with and without diabetes. NT-proBNP levels were not influenced by the presence of diabetes, whereas Tn-T levels were elevated in HF patients with diabetes. Diabetes, high NT-proBNP and high Tn-T were highly predictive for CV death or HF hospitalization (55).

The above-mentioned studies show a combined prognostic value of using cTn with BNP or NT-proBNP for predicting HF morbidity and mortality, as is also mentioned in the ESC and the AHA guidelines (16, 17). Comparable to NT-proBNP, cTns are cardiac-specific, which provides them with high tissue specificity. At the same time, latest advances in improving high and ultra-high sensitive assays has largely improved sensitivity of cTn detection. This has lately opened new approaches to evaluate cTn as a prognostic biomarker even in the general population, with detectable values in nearly 100% of healthy individuals (56). These new opportunities to evaluate cTn as a marker for subclinical cardiac pathologies seems promising. On the other hand, such discrete elevations of circulating cTn levels may merely represent higher protein turnover. Patho-mechanistically, cTns are passively released into the blood flow upon myocyte injury as opposed to NT-proBNP which is actively secreted upon pathological triggers in early cardiac disease states. This could make NT-proBNP the better, more sensitive, cardiac-specific HF prognosis biomarker—independent of the sensitivity of the used assay. Nevertheless, hs-cTn assays have been proven beneficial since they allow detection of Tn in patients with stable chronic HF in the absence of acute myocardial damage (57) and could aid in improving HF prognosis, when used additionally to NT-proBNP in a multi-biomarker approach.

### Cardiac Myosin-Binding Protein C

Cardiac myosin-binding protein C (cMyBP-C), encoded by the *MYBPC3* gene, is a component of the sarcomere thick filament complex along with titin and myosin. cMyBP-C plays an important role in sarcomere structure and maintenance, and regulation of muscle contraction through modulation of actin-myosin cross-bridges (58, 59). cMyBP-C is a large modular protein of 140.8 kDa with 11 globular domains and belongs to the intracellular immunoglobulin (Ig) superfamily (60, 61). cMyBP-C differs from the skeletal isoform sMyBP-C, in that cMyBP-C contains 3 additional domains: C0 (at the N-terminus), C1 and C2. The region between C1 and C2 domains contain functionally important phosphorylation sites, which confer cardio-protection and reduce cMyBP-C association to actin and myosin (60, 62–64). After an event of MI, phosphorylation of cMyBP-C results in the release of a cleaved ~40 kDa N-terminal fragment of cMyBP-C into the circulation (65–67). This phosphorylation protects cMyBP-C from proteolysis during ischemic injury and can therefore serve as a diagnosis biomarker for cardiac injury upon hospital admission (65, 68). Phosphorylation by phosphatase kinase A (PKA) is important, because reduction in phosphorylation of sarcomeric target protein c-MyBP-C has been reported in end-stage failing myocardium irrespective of the cause of HF (69–73).

As a circulating biomarker, cMyBP-C has first been assessed as a highly sensitive marker for myocardial injury and recent studies provide evidence that cMyBP-C could outperform cTn in the early detection of MI (74–77). Reasons for cMyBP-C's earlier detectability in the circulation could be its higher abundance in cardiomyocytes compared to cTn (74) and, more importantly, an ischemia-induced shedding of cleaved N-terminal fragments of cMyBP-C (78). These cardiac-specific and highly sensitive characteristics make circulating cMyBP-C an ideal target to be evaluated as a biomarker for subclinical CVD states and also a candidate biomarker for HF prognostication. In a prospective case-control study involving 50 children with acute HF, cMyBP-C levels were measured at the time of hospital admission and 1 month after treatment with a follow-up at 3 months. The authors reported that higher levels of cMyBP-C at the time of hospital admission were associated with worse prognosis and higher rate of readmission and mortality (79). Surprisingly, these promising initial results in HF outcome prognostication have so far not been re-assessed. Only one further study assessed cMyBP-C in early subclinical CVD disease states; Anand et al. reported cMyBP-C to be associated with myocardial hypertrophy and fibrosis, as potential causes for HF, in aortic stenosis patients (80). The authors furthermore report an association of cMyBP-C with mortality in the assessed cohort.

cMyBP-C is an important regulatory protein of the cardiac contractile complex. As a biomarker, there is convincing data on its promising potential to be clinically used to improve early rule-in and rule-out of MI. With respect to HF prognostication further trials are warranted to validate the prognostic and diagnostic ability of cMyBP-C.

### Heart Type Fatty Acid Binding Protein

Heart type fatty acid binding protein (hFABP), encoded by the fatty acid-binding proteins 3 (*FABP3*) gene, is a member of cytoplasmic protein group and is classified as a cardiac cell death marker (81). In general, FABPs play a role in cellular fatty acid metabolism by binding to and transporting long-chain polyunsaturated fatty acids from the cell membrane to mitochondria (82). hFABP is a 15 kDa protein, expressed abundantly in the cytoplasm of striated muscle cells. Unlike Tn or cMyBP-C, hFABP is not a component of muscle structure (83–86). Therefore, it is likely that hFABP is released immediately upon cardiomyocyte injury, as in case of MI, and may be detectable earlier than cTn and cMyBP-C. Importantly, the cytosolic localization of hFABP could make an important patho-mechanistic difference in terms of active secretion as opposed to passive release after cardiomyocyte injury. This may provide a different quality of information on cardiac pathology in terms of early detection of subclinical disease or HF prognostication. hFABP starts a negative cycle of cardiac damage because increased extracellular hFABP levels affect cardiac contraction by decreasing intracellular calcium levels, therein causing further damage and more extracellular hFABP levels (82). hFABP levels in serum rise immediately after cardiomyocyte injury, making it a promising molecule to investigate with respect to cardiac function and HF, although the concentration variability of hFABP in HF patients is less well known.

Kazimierczyk et al. investigated the prognostic ability of hFABP in 77 patients with acute decompensated HF at hospital admission and discharge. The authors reported that constantly higher levels of hFABP might reflect ongoing myocardial damage and might be a valuable biomarker to predict poor outcome in acute decompensated HF patients (87). A study by Niizeki et al. investigated serial measurements of hFABP levels in 113 chronic HF patients at the time of hospital admission and at the time of hospital discharge. The patients with consistently high levels of hFABP had subsequent higher cardiac events in the follow-up period (624 ± 299 days) when compared to patients with normal hFABP levels or those, whose levels decreased between admission and discharge. The authors concluded that such serial measurement of hFABP can be informative for guiding therapy and management of chronic HF patients (88). Another study by Niizeki et al. investigated whether the combination of hFABP and BNP would provide information on risk stratification in 186 chronic HF patients. High hFABP and BNP levels at the time of hospital admission were associated with increased number of cardiac events and mortality, therefore were helpful in risk stratifying chronic HF patients upon hospitalization (89). A *post-hoc* analysis of the MANPRO study (2015) by Hoffmann et al. investigated the prognostic ability of hFABP when compared to Tn-I in patients suspected of acute HF with a 5-year follow-up period. Higher hFABP levels were associated with all-cause mortality and acute HF related hospitalization at 5-years, and hFABP levels could predict the acute HF related hospitalization better than Tn-I (90). In a study by Kutsuzawa et al. (91), hFABP and Tn-T levels were measured in 151 prospectively enrolled HFpEF patients. Higher levels of hFABP was observed more frequently in patients when compared to circulating Tn-T levels, indicating that cardiomyocyte membrane injury occurred frequently compared to myofibrillar damage in HFpEF patients. Circulating levels of hFABP increased with advancing NYHA functional class and was an independent predictor of future CV events (91).

The above reports suggest hFABP as a biomarker for CVD, not primarily focusing on MI like cTn and cMyBP-C. Instead, circulating hFABP may well be a marker of cardiomyocyte-specific metabolic disorders as they occur not only during but also before the onset of HF, thus making hFABP a promising candidate biomarker for very early stages of subclinical HF, and for HF prognostication. On the other hand, currently available data is limited to a low number of trials and large-scale validation is needed in order to gain more information on its potential for clinical implementation.

### Galectin 3

Galectin 3 (Gal-3), encoded by *LGALS3*, is a member of the galectin family and is a beta-galactosidase-binding lectin with an atypical N-terminal domain (92). Gal-3 is ubiquitously expressed and plays an important function in several biological processes such as in cell-cell adhesion, cell-matrix interactions, macrophage activation, angiogenesis, metastasis, and apoptosis (92). Hence, Gal-3 is not cardiac-specific. Basal Gal-3 expression varies depending on tissue type and tissue maturity (93). Within hematopoietic tissue, macrophages express Gal-3 more than monocytes and it is undetected in human peripheral blood lymphocytes (94–97). This increased expression of Gal-3 in macrophages is assumed to induce inflammation, fibroblast proliferation, and collagen deposition in the heart, thereby promoting ventricular restructuring, which is a central patho-mechanistic process in HF (98–100).

In the CORONA study, the prognostic value of Gal-3 was tested in 1,462 patients aged >60 years with systolic, ischemic HF. Using an unadjusted analysis, Gal-3 was not associated with HF hospitalization but with CV death. When adjusted for NT-proBNP, Gal-3 showed no significant association with any end-points. The authors concluded that prognosis of HF in elderly patients with systolic HF is limited using Gal-3 (101). In the HF-ACTION study, the association between Gal-3 and long-term clinical outcome in ambulatory HF patients was evaluated. In a univariate analysis, Gal-3 was significantly predictive of long-term outcomes [unadjusted hazard ratio, 1.14 (per 3-ng/mL increase in Gal-3), 95% CI 1.09–1.19; *p* < 0.0001], however this association did not withstand multiple testing or adjustment for NT-proBNP (adjusted hazard ratio: 1.03, 95% CI 0.98–1.08, *p* = 0.27) (102). Conversely, there are studies or analyses which have shown that Gal-3 can be helpful in predicting adverse outcomes when measured serially (baseline and follow-up). In the DEAL-HF study (2010), Gal-3 was measured in 232 patients with chronic HF (NYHA class III or IV) at baseline and 6.5-year follow-up. Plasma levels of Gal-3 proved to be a significant predictor of mortality (103). In two large cohorts of chronic HF and acute decompensated HF patients, the prognostic value of Gal-3 serial measurements in HF patients at baseline vs. 3-months follow-up (CORONA study) and baseline vs. 6-month follow-up (COACH study) revealed that an increase of Gal-3 at follow-up was associated with an increased rate of re-hospitalization and mortality (hazard ratio in CORONA, 1.60; 95% CI, 1.13–2.25; *p* = 0.007; hazard ratio in COACH, 2.38; 95% CI, 1.02–5.55; *p* = 0.046) (104).

The above information suggests that Gal-3 may well be a biomarker in the setting of HF. Its expression is upregulated in fibrosis, a common phenomenon associated with HF. The tissue of origin remains a matter of debate and thus its lack of cardiac specificity seems to be reflected by a limited prognostic value when compared with more established biomarkers such as NT-proBNP.

### Growth Differentiation Factor 15

Growth Differentiation Factor 15 (GDF15), also known as macrophage inhibitory cytokine-1 is a distant member of the transforming growth factor-β cytokine superfamily (105–107). The secreted form of GDF15 is a ~28 kDa disulphide-linked dimer that is expressed in low levels in all organs except the placenta (105, 108). More specifically, GDF15 expression arises from macrophages, vascular smooth muscle cells, endothelial cells and adipocytes (109–114). An increase in GDF15 levels is observed under cardiac inflammation, injury, and restructuring (115–117). GDF15 was detected in atherosclerotic plaques in coronary arteries (109, 118) although the origin tissue of GDF15 expression in HF remains unclear.

GDF15 biomarker measurements in HF patients has provided evidence of its capability in HF prognosis, either through serial measurements or when measured along with other prominent biomarkers. In the Valsartan Heart Failure Trial (Val-HeFT), the investigators evaluated serial measurements of GDF15 at baseline (*n* = 1,734) and 12-months follow-up (*n* = 1,517) in HF patients. GDF15 levels were independently associated with risk of death, even after adjustment for BNP, hs-Tn-T, and hs-C reactive protein. Serially increasing GDF15 levels at follow-up were associated with worsening renal function and increase in cardiac strain biomarkers (BNP, Tn-T) (119). In another study, elevated levels of GDF15 were associated with increased risk of death in 455 chronic HF patients. Even after adjusting to NT-proBNP and other markers, GDF15 retained the prognostic ability in predicting HF mortality (120).

HF causes release of GDF15, whereas the specific tissue of origin is not completely determined. Instead, the utility of GDF15 must be taken as marker for systemic causes or effects of HF. Whether GDF15 allows for specific HF-related prognostication remains matter of debate. We can speculate that in patients with other causes of systemic inflammatory processes, GDF15 may lose its HF-specific prognostic value. Nevertheless, results from clinical trials suggest a promising role of GDF15 as a biomarker in HF prognostication. In this respect, further investigations and validations remain to be undertaken.

### Soluble Suppression of Tumorigenicity 2

Soluble suppression of tumorigenicity 2 (sST2), is a member of interleukin (IL)-1 receptor family and is a ligand for IL-33 (121). IL-33 is an IL-1 like cytokine that can be secreted by most cells in response to damage (122). Whereas, sST2 is produced by vascular endothelial cells, cardiomyocytes, and cardiac fibroblasts in response to stress or injury providing a certain degree of cardiovascular specificity. sST2 is the circulating isoform of the cellular membrane receptor ST2L, lacking the cytoplasmic, and transmembrane domains. IL-33/ST2L is beneficial to the heart because it inhibits cardiac hypertrophy and fibrosis, thereby mitigating adverse cardiac remodeling (123). This beneficial effect is blocked by increased levels of sST2 (acting as decoy receptor) (123), since sST2 binds to IL-33 and interrupts the IL-33/ST2L downstream signaling.

Serial measurement of sST2 at baseline and at follow-up provides evidence of the prognostic impact of sST2 in predicting HF hospitalization outcomes. In the ASCEND-HF trial, serial measurement of sST2 levels at baseline and follow-up at 48, 72 h, and 30 days were investigated in 858 acute HF patients for adverse HF outcomes. Continuously higher levels of sST2 was associated with increased risk of adverse HF events and higher risk of death at 180 days [hazard ratio at baseline: 2.21 (*p* < 0.001); at follow-up: 2.64; (*p* < 0.001)]. However, the prognostic value of sST2 decreased after adjustment with NT-proBNP (124). In the MADIT-CRT trial, sST2 levels were serially measured at baseline and 1-year follow up in 684 patients with mild symptoms of HF and reduced LV function. Elevated sST2 levels at baseline was associated with increased risk of death, HF or ventricular arrhythmia (VA) events, and serially increasing levels of sST2 was associated with higher risk of VA and death (125). In the Valsartan Heart Failure Trial (Val-HeFT), sST2 was serially measured at baseline, 4 and 12-months follow-up in patients with HF. Increased sST2 levels were independently associated with morbidity, mortality and HF hospitalization. However, it failed to provide prognostic information when adjusted with NT-proBNP (126).

The IL-33/ST2L system exerts cardio-protective function, which is interrupted by sST2's decoy receptor capabilities. Circulating sST2 can provide information about cardiac stress and can be targeted pharmaceutically to maintain the IL-33/ST2L cardio-protective role. Clinical evaluation of circulating sST2 provide initial data on its use as a prognostic biomarker in HF, albeit its additional values compared to more cardiac-specific markers remains limited and subject to further large-scale investigation.

## Genetic Biomarkers

**Figure 3:**
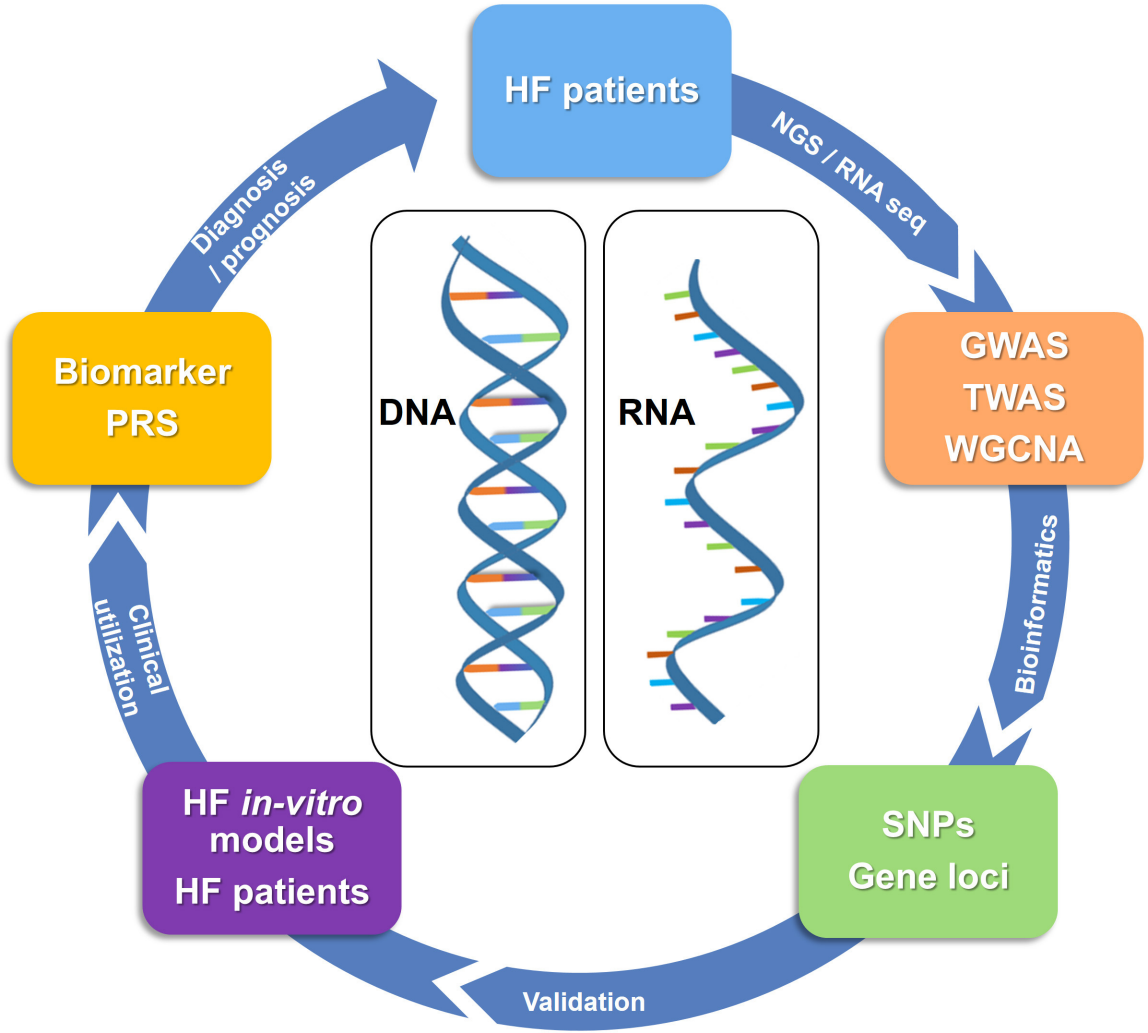
Identification and validation of genetic biomarkers in HF. In order to identify genetic biomarkers, samples taken from HF patients are sequenced and analyzed via GWAS, TWAS as well as WGCNA. Results are then further processed using bioinformatics providing information on SNPs or gene loci associated with HF. Validation of these SNPs by *in-vitro* models for HF and HF patients provides concrete evidence on the clinical utilization of genetic biomarkers or PRS. Ultimately, genetic biomarkers can be used for diagnosis/prognosis of HF patients in the future. DNA, deoxyribonucleic acid; GWAS, genome-wide association studies; NGS, next generation sequencing; PRS, polygenic risk score; RNA, ribonucleic acid; RNA seq, RNA sequencing; SNPs, single nucleotide polymorphisms; TWAS, transcriptome-wide association studies; WGCNA, weighted gene co-expression network analyses.

Currently, the clinical standard for prognosis for HF depend on protein-based biomarkers as they bear many advantages such as easy accessibility, comparably low costs, and easy handling. Nevertheless, protein-based biomarkers might not be specific for HF and therein definite prognostic markers for HF remain scarce (127). This scarcity is partly caused by the complex and diverse etiology and pathophysiology of HF (1, 128). Recently, new opportunities for genetic analyses have risen as a novel approach to understanding the pathophysiology of CVD, paving the way for the development of gene-based biomarkers. Omics technology that identifies genome-wide (GW) and transciptome-wide (TW) gene variation is an innovative approach to identify DNA/RNA-based biomarkers. Omics analyses not only allow to identify genetic variations that can contribute to at-risk HF identification but also for understanding the molecular mechanisms behind the diseases. By identifying a common single nucleotide polymorphism (SNP) (through GW) or gene expression variation (through TW), it is possible to stratify at-risk populations for a disease. This brings us one step closer to personalized medicine (Figure 3). It has to be noted, however, that given the origin and novelty of these analyses and the complexity of CVD, most analyses broadly consider CVD and only few limited studies are available specifically for HF. Although it was well-established that there is a genetic component for the development of CVD (129), the causal role of genetics in the development of CVD remained largely elusive until genome-wide association studies (GWAS) became available. In 2007, four groups discovered the 9p21 risk locus simultaneously by using GWAS analyses (130–133). The locus encodes different transcripts of the long non-coding RNA ANRIL (134). Current studies suggest that the ratio of circular to linear ANRIL, which affects basic cellular mechanisms, is associated with the risk of coronary artery disease (CAD) and could potentially serve as a biomarker (135). Subsequently after discovery of the 9p21 locus, multiple genetic loci were identified that, all together, account for ~25% of the estimated CVD heritability (135). These results changed the understanding of the genetic architecture of CVD where instead of rare variants of SNPs having large effects on CVD risk in most patients, the genetic risk for CVD derives from the cumulative effect of many common risk alleles, each of them with small effect sizes (135).

### Genetic Biomarkers Combined With Lifestyle Risk Factors

As HF is a multi-causal disease, risk scores integrating clinical diagnostics, protein, and genetic biomarkers as well as lifestyle factors bear a huge potential for personalized risk stratification and prevention strategies for HF. In this regard, easily accessible biomarkers for lifestyle risk factors like obesity and smoking can also be an important approach for objective risk assessment and the identification of molecular mechanism for the development of HF. This in turn might lead to the identification of novel HF biomarkers as well. Adipokines like leptin and chemerin are examples of protein-based markers derived from the adipose tissue. They are not only obesity-associated but also dysregulated in cardiovascular diseases (136, 137). For smoking, a number of affected loci have been identified by genetic and epigenetic analyses, including the *AHRR, GPR15, GFI1*, and *RARA* genes, which in turn might be involved in cardiovascular diseases (138, 139). The bioinformatics integration of these data and validation in large cohorts can result in a biomarker panel for risk stratification. For HF prognosis, genetic analyses bear the potential for future identification of genetic and protein biomarkers for early as well as late HF prognosis, thereby improving personalized treatment. As a future scenario, a panel of protein-based and genetic-based biomarkers for inheritable and lifestyle factors might be measurable from just one single blood draw, which altogether integrate into a highly accurate HF risk score.

### Genome Wide Association Studies (GWAS) and Weighted Gene Co-expression Network Analyses (WGCNA)

Currently, more than 160 chromosomal loci associated with CAD risk have been identified by GWAS consortia. These loci affect pathways such as lipid metabolism, blood pressure, inflammation, blood coagulation, cell cycle and proliferation, signal transduction, apoptosis, and transcription splicing regulation (135, 140). Fewer gene loci have been identified specifically for HF in contrast to the large number of GWAS loci associated with CAD risk. An overview on HF-specific genomic loci identified by GWAS analyses is given by van der Ende et al. (141). The likely affected genes comprise *KIAA1598, USP3, LRIG3, HSPB7, BAG3*, and *HCG22* (141). Interestingly, *USP3, LRIG3*, and *HSPB7* have also been linked to HF in other studies. *USP3* codes for the ubiquitin specific peptidase 3, which might be involved in the development of HF (141). *LRIG3* has previously been connected to several types of cancers, but Lrig3 knockout in mice also impaired cardiac function (142), *HSPB7* encodes the heat shock protein B7, which is mostly expressed in cardiac and skeletal muscle and preserves contractile integrity. It is also referred to as the cardiovascular heat shock protein (cvHSP) and has been associated with advanced HF (141, 143). A recent study by Shah et al. combining a GWAS meta-analysis with Mendelian randomization analysis identified eleven HF-associated loci, among them another variant within the above mentioned *BAG3* locus (128). *BAG3* encodes the B cell lymphoma 2-associated anthanogene protein, which is an anti-apoptotic co-chaperone protein (144). It plays an essential protective role in dilated cardiomyopathy and is associated with LVEF (144–146). These genes and proteins are interesting candidates for novel prognostic markers for HF and their role and mechanisms should further be experimentally elucidated. An example for a potential GWAS-derived HF biomarker is the SNP rs12564445 within the *TNNT2* gene which encodes the cardiac troponin T protein. The SNP rs12564445 was associated with incident HF in European Americans (147). As described in this review, cardiac troponin is clinically used as a protein-based biomarker for the diagnosis of HF. Furthermore, cytokine gene polymorphisms, e.g., in the Interleukin 10 (*IL-10*) gene might act as biomarkers to identify individuals more susceptible to HF (148). *IL-10* is an anti-inflammatory cytokine with pleiotropic effects in immune regulation. Experimentally, *IL-10* has been shown to act in a cardio-protective way and, amongst other mechanisms, antagonizes TNF-α, which is an important cytokine for heart failure progression (149). Given its manifold effects, it has to be further evaluated whether *IL-10* can be used as a heart failure-specific biomarker in the future. Hypertrophic cardiomyopathy (HCM), a disease of the sarcomere, is identified by mutations in *MYBPC3* gene encoding cMyBP-C. More than 350 mutations have been identified in the *MYBPC3* gene, representing 40–50% of all HCM mutations, making it the most frequently mutated gene in HCM disease (150, 151). More than 60% of *MYBPC3* mutations are truncating, slicing, or branch point mutations leading to COOH-terminally truncated cMyBP-C protein that lacks major myosin- and/or titin- binding sites (151).

As a step further than GWAS, weighted gene co-expression network analyses (WGCNA) allow functional interpretations of gene network modules (152). In a recent WGCNA analyses, the six hub genes *BCL3, HCK, PPIF, S100A9, SERPINA1*, and *TBC1D9B* were identified in HF patients after acute MI and could potentially serve as early prognostic biomarkers for HF (152). These hub genes might be involved in the development of HF by regulating local and systemic inflammatory pathways (152). For instance, *BCL3* encodes the proto-oncogene B-cell lymphoma 3-encoded protein (153). Amongst others, *BCL3* is involved in the transition from compensated cardiac hypertrophy to HF (154). Even though these hub genes and many other genetic risk loci have been described in relation to HF and CVD, their translation as biomarkers into the clinic remains difficult as described below. The longest known genetic CVD biomarkers that were translated into the clinic include mutations in the *LDLR, PCSK9*, and *APOB* genes, which can lead to hypercholesterolaemia (135). Screening for these mutations gives the opportunity for early diagnosis and personalized treatment with lipid-lowering medication (135). Although novel genomic analyses have not led to genomic biomarkers yet, transcriptome analyses were the means for identification of protein-based biomarkers like GDF15 and ST2. GDF15 has been identified by gene expression array in cardiomyocytes under nitrosative stress and ST2 was identified by microarray technology to be upregulated in cardiac myocytes after mechanical strain (115, 155).

### Polygenic Risk Scores

The identification of CVD loci has not revolutionized diagnostics or treatment of CVD yet because CVD is a complex multi-genic disease and SNP variants have mainly small effect sizes. As strong genetic biomarkers have not been established for HF, genetic risk scores were a new approach to consolidate the small effect sizes of SNP variants. Initial studies creating genetic risk scores had only limited success due to small size of the initial GWAS, limited computational methods and lack of large datasets for validation and testing (156). Presently, polygenic (multiple genes) risk scores (PRS) include a large number of genetic variants that are able to identify more people at risk for CVD, as it has been shown to be possible with rare monogenic mutations that appear to be the main cause for positive CVD family history (156, 157) In 2018, Khera et al. published a PRS, wherein 8% of the population were identified to have a genetic predisposition, resulting in a more than 3-fold increase in CVD risk (156). Participants with a high genetic risk score would benefit from a 50% lower risk of CVD by adhering to a healthy lifestyle (158). Furthermore, another current clinical study combined a PRS for CAD with lifetime exposure of LDL-C and systolic blood pressure (159). This study concluded that although the PRS categorizes the lifetime risk for CAD, modifiable risk factors are the main influence for the development of the disease (159).

Presently, few gene panel assays are commercially available but have not been applied in clinical settings (160, 161). Even though PRS were able to identify individuals at risk for CAD, it did not add significant predictive value compared to traditional risk factors and the clinical relevance of PRS might be less than expected (162, 163).

### Challenges and Prospects of Using Genetic Biomarkers

Though many genetic loci for increased CVD risk have been identified, the heritability of CVD is still largely unknown. One reason is that the functions of the identified loci remain largely elusive. Only 30% of the SNPs were found to modify classic risk factors for CVD and very few affect the protein structure (140). Most variants are located within non-coding regions, which impedes the identification of their function or targets. Furthermore, most of the loci affect multiple genes and phenotypes, making it difficult to identify the causal variant (135). Additionally, CVD is a complex heterogeneous disease and genetic analyses mostly did not consider environmental factors, the various triggers for HF and were limited to CAD/CVD patients of European ancestry (156, 164, 165). Therefore, performance of PRS and the sensitivity and specificity of genomic loci as HF prognosis biomarkers needs to be validated in different large-scale studies as well as in different ethnicities (166, 167). Large-scale population-based cohort studies are required to allow for the evaluation of a broad range of phenotypes together with high-density genetic mapping.

Presently, the laboratory methodology for genetic biomarkers is time-consuming, complex, and expensive for clinical routine compared to protein-based biomarkers and currently these genetic biomarkers do not add significant additive value to classic cardiovascular risk factors. However, in a mid- to long-term perspective, genetic biomarkers may provide a unique opportunity for treatment and early risk prediction through lifestyle changes or medication. If genetic biomarkers are combined with classic and clinical risk factors, they can add significant value by helping clinicians to practice personalized medicine at an early point of the disease process (159).

## Transcriptomics Based Biomarkers—Non-coding RNAs

**Figure 4:**
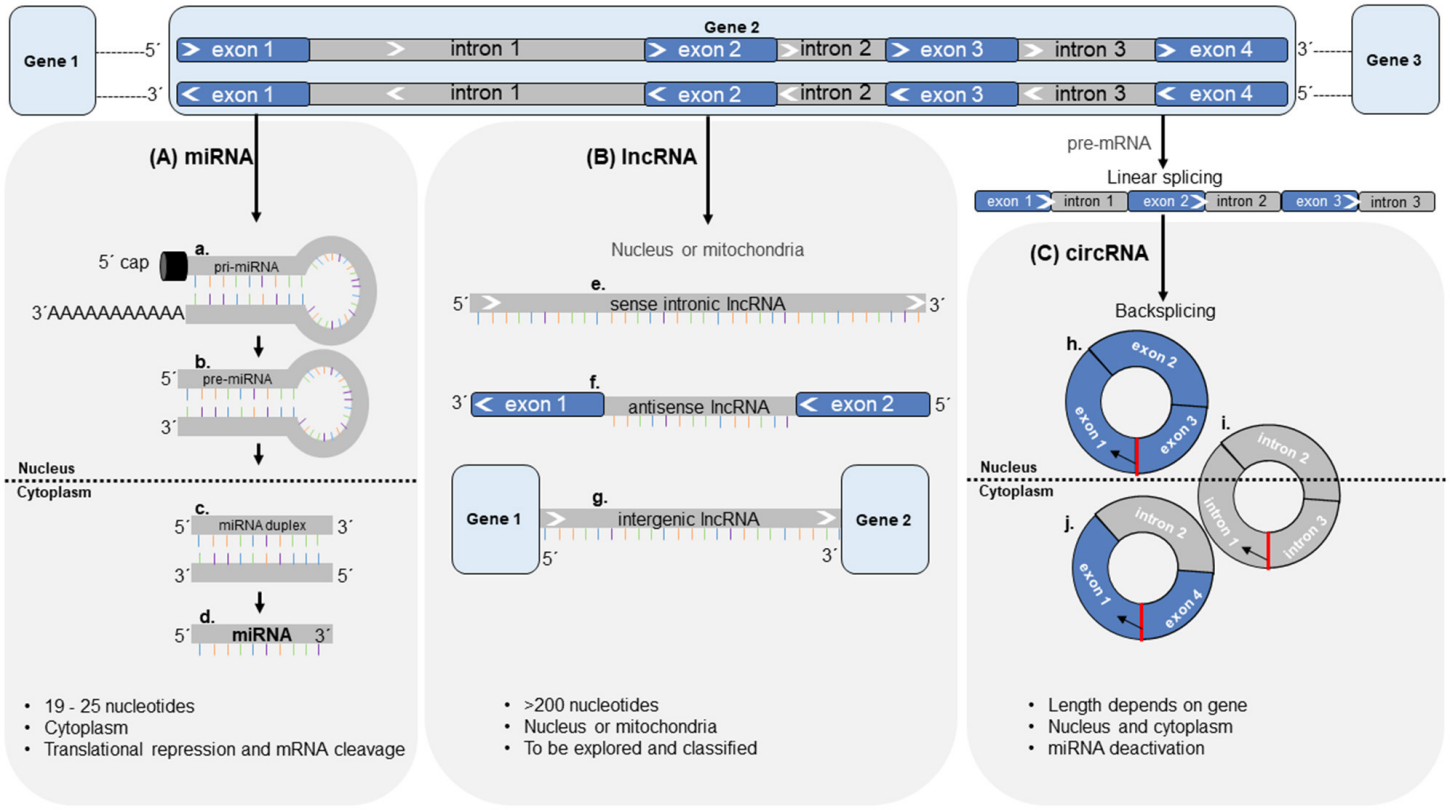
Synthesis, length, cellular location and molecular function of non-coding RNAs. **(A)** miRNAs are expressed from miRNA genes. Located in the nucleus, pri-miRNAs **(a)** are transcribed from the DNA and are processed by Drosha-Pasha processing complexes giving rise to pre-miRNAs **(b)**. Pre-miRNAs are translocated from the nucleus into the cytoplasm via Exportin-5, where they are further processed by Dicer enzyme to form miRNA duplex **(c)** and later the final miRNA **(d)**. **(B)** lncRNAs arise from sense and antisense strands of introns **(e)**, exons **(f)**, and intergenic regions **(g)** of the DNA and are located within the nucleus or mitochondria. **(C)** circRNAs are generated from the pre-mRNA in a process called back-splicing' resulting in circRNAs made up of either exon-exon junctions **(h)**, intron-intron junctions **(i)** or exon-intron junctions **(j)**. Red indicates the back-splice junction. Black arrows indicate direction of back-splicing in circRNAs. circRNA = circular RNA, lncRNA = long non-coding RNA, miRNA = microRNA, mRNA = messenger RNA.

Transcripts of non-coding regions in the genome have been widely implicated in biology. Several subcategories of non-coding RNAs have been described (Figure 4). Of these, most attention has been focused on microRNAs (miRNAs) since their discovery in the early 1990's (168). Over the last decade, other classes have been identified, such as long non-coding RNAs (lncRNAs) and circular RNA (circRNAs). Several of these have been studied in the context of cardiovascular disease (CVD). Most strikingly, several subclasses of ncRNA are readily detected in the circulation (169)—freely circulating as well as associated with and derived from circulating cells such as leukocytes and platelets. This has opened up new avenues of biomarker research, which has traditionally relied on protein measurements (78).

### miRNAs

miRNAs are an abundant class of non-coding RNAs of 19–25 nucleotides in length, recognized as evolutionarily conserved RNA molecules that fine-tune protein synthesis by regulating gene expression at the post-transcriptional level (Figure 4). By binding to the 3′-untranslated region (UTR) of messenger RNAs (mRNAs) through sequence complementarity, miRNAs either initiate translational repression or mRNA degradation. For some miRNAs, expression is confined to a specific organ or cell type (170–172) and thus, these miRNAs are expressed in a tissue- and cell-type specific fashion (173, 174). Intracellular miRNAs have been proven to be important modulators of cell function under pathological conditions (175). A single miRNA can suppress more than one gene. Conversely, single genes can be targeted by multiple miRNAs in parallel. Targets of individual miRNAs are often found to be functionally related. Gene suppression is usually partial rather than total, indicating that miRNAs fine-tune protein levels (169).

miRNAs are not just confined to the intracellular space of organ tissues, but also present in the circulation (176) within circulating cells such as platelets and as a pool of extracellular miRNAs. The stable detection of these miRNAs was surprising, given the high levels of RNase activity in blood plasma. In contrast to mRNA, miRNAs are protected from instant degradation through several mechanisms (177–179). Circulating miRNAs were initially thought to be a passive spill-over from various tissues. However, there is emerging evidence for their function as extracellular messengers in cell-to-cell communication. This might provide an alternative pathway of gene regulation in physiological and disease conditions (180), although the biological relevance of this phenomenon remains to be matter of debate (181). Independent of a biological function of circulating miRNAs, their potential use as circulating biomarkers for CVD has been increasingly recognized over the past years (182, 183). *In vivo* findings suggest that specific subsets of miRNAs are dysregulated with different features of CVD (184) and several studies have explored their potential clinical utility.

Most miRNAs are ubiquitously expressed across tissues. The earliest reports in the cardiovascular field were published in 2005, identifying miR-1 as a regulator of cardiac differentiation (185, 186). miR-1 plays a key role in the differentiation process of mesodermal precursors to cardiomyocytes and is involved in reprogramming cells into cardiomyocytes (187). Furthermore, miR-1 restoration in failing hearts improved cardiac function (188), suggesting not only its essential biological role but pointing out miR-1′s potential as a therapeutic target in heart failure treatment. Patho-mechanistically, cardiac remodeling and fibrosis are essential and causal pathological processes in the development of HF and there is proof that miRNAs are key regulators in developing and sustaining remodeling and fibrosis (172). In fact, miR-1 was identified as downregulated in an *in-vivo* mouse model of induced cardiac hypertrophy via regulation of calmodulin and Mef2a—both known to be hypertrophy-associated (189). In cardiac tissue of n=34 HF patients mir-1, among other miRNAs, was upregulated compared to healthy controls (190). When assessed as a circulating biomarker, in a highly controlled setting of doxorubicin-induced heart failure in *n* = 56 female patients with breast cancer, miR-1 was identified as a potential prognostic biomarker to identify individuals who later on develop a reduction in EF (191). Although derived from a small cohort, these results are particularly interesting when comparing the AUC of 0.851 for miR-1 with that of cTn (0.544). These results were validated in n=49 MI patients in which miR-1 was reported to negatively correlate with ejection fraction, suggesting miR-1 as a predictive biomarker for HF onset after MI (192). Other reports were not able to validate these findings (193), which may be caused by a number of different potential reasons: (1) the detectability of cardiac miRNAs in the circulation is generally low unless measured in samples of patients suffering from severe MI (74), introducing a potential detectability bias in quantification efforts; (2) up to date there are no harmonized methods with respect to miRNA quantification, which is particularly problematic for miRNA quantification in low-RNA-yield samples such as plasma and serum in terms of comparability of the results (78); (3) Only few large-scale studies have been conducted assessing the prognostic properties of miRNAs in CVD and their common interpretation of the results is, that not single miRNAs, but instead miRNA combinations comprise prognostic potential as biomarker (194, 195).

Detailed reviews of specific cardiac-enriched (187) and other miRNAs (196) involved in HFrEF and HFpEF (172) are beyond the scope of this article and can be found elsewhere. Using miR-1 as an example, it can be seen that miRNAs are important biological players in the development of HF and are suggested as promising circulating biomarker in HF prognostication, while on the other hand validation of initial findings is pending and methodological issues remain to be solved.

### lncRNAs

Further to miRNAs, long non-coding RNAs (lncRNAs) were recently investigated as regulators of protein function (197). lncRNAs are a broad group of RNA, >200 nucleotides in length (198). There is thus far no agreement on sub-classification for these RNAs. A recent review summarized different characteristic features that could be used for this purpose, comprising of their length, their relation to protein-coding genes or their relation to DNA/promoter elements (199). Unlike miRNAs, lncRNAs are mainly located within the nucleus or in mitochondria (200, 201) (Figure 4) and their biosynthesis seems to largely overlap with that of mRNAs with regards to their transcription, polyadenylation, capping, and splicing (202). For the majority of identified lncRNAs, the function remains unclear. Nuclear lncRNAs are involved in regulation of neighboring loci through transcriptional regulation or by inhibiting expression of a gene through sequestration of transcription factors (203). Conversely, other lncRNAs were shown to enhance transcription of genes. lncRNAs are more tissue-specific than protein coding genes (200) and compared with miRNAs, many more transcripts have been identified (204). They have emerged as mediators of protein translation (205). Data is available suggesting key regulatory roles of lncRNAs in cardiac and vascular tissue with respect to CVD (205). Both, miRNAs and lncRNAs are potent regulators of translation and their expression influences protein levels, while at the same time these two ncRNA species influence each other's expression (206).

Several lncRNAs are also readily detected in the circulation. This indicates the presence of protective mechanisms against RNase-mediated degradation, for which the mechanisms show overlap with those of miRNAs (198). The plasma level of Long Intergenic ncRNA Predicting CArdiac Remodeling (LIPCAR) was found to predict adverse cardiac remodeling and death in the aftermath of MI, imposing an increased risk for ischemic cardiomyopathy and HF (201). Thus, LIPCAR has potential as a circulating biomarker for HF prognostication, but has not yet been evaluated in a clinical trial. Myosin Heavy Chain Associated RNA Transcripts (MHRT) levels were found dysregulated in plasma depending on the SNP alleles (rs7140721, rs3729829, and rs3729825) in chronic HF patients. Significant difference in risk of mortality was observed based on these SNP genotypes (*p* < 0.001) indicating an association of these SNPs with chronic HF risk and prognosis (207). The latter might provide a way to link a single circulating molecule/biomarker with genetic risk prediction, while additional evaluation of this interesting link remains to be further explored. lncRNA H19 was discovered to be down-regulated in failing hearts from mice and was validated in pig and human hearts (208). The authors were further able to prove H19's essential HF-reversing effect. While these findings are backed-up by similar results on cardiac tissue level (209), validation of H19 as a circulating biomarker for HF prognostication is still pending. These findings indicate the potential use of lncRNAs as prognostic circulating biomarkers for CVD—similar to some miRNAs.

lncRNAs are promising RNA molecules with good characteristics as circulating biomarkers for CVD such as detectability in the circulation and distinct biological function in the heart. Their general exploration as circulating biomarkers is still in its infancy and more interesting results can be expected in the near future.

### circRNAs

The first single-stranded DNA product (replicating form of DNA) that was shown to have a circular shape was described by Chandler et al. in 1964 (210), whereas the first circular RNA was described a decade later in plant viroids (211). Before circular RNAs were first described in humans in 1993, RNA species were identified, where “*exons were joined accurately at consensus splice sites, but in an order different from that present in genomic DNA*”(212). These “scrambled exons” were described as stable and situated in the cellular cytoplasm (213). Only during the past decade however, novel RNA analysis tools such as biochemical enrichment strategies and high-throughput deep sequencing methods have allowed for large numbers of circRNAs to be detected (214). circRNAs are a stable RNA species, endogenous to mammalian cells and proven to be expressed in a tissue- and developmental-specific context (214, 215). They can either emerge from exons or introns of primary gene transcripts (pre-mRNA) (215, 216) and are products of alternative splicing in a head-to-tail fashion known as “back-splicing” (214) (Figure 4). circRNAs are resistant to degradation by the exonuclease RNase R—a type of RNase that cleaves linear RNA. RNase R treatment can therefore be used to enrich circRNAs over their linear counterparts (217, 218). In combination with the use of divergent primers in polymerase chain reaction (PCR) amplification, this approach yields high specificity for the detection of circular transcripts.

Functionally, circRNAs appear to influence gene expression in different ways. They act as potent miRNA sponges—decreasing the inhibitory effect of miRNAs on protein synthesis (219). More recently, circRNAs were reported to be translated into proteins (220). At the same time, their expression is regulated by proteins such as RNA-binding proteins. circRNAs appear to influence gene expression by competing with splicing of their linear counterparts (173, 218, 221). circRNA expression has been mapped in different tissue types and it is now clear that they can be reliably detected in a tissue- and cell-specific manner, whilst also showing a certain degree of conservation across species (173, 222).

Bearing in mind the vast opportunities for disease detection and possibly treatment offered by miRNAs, efforts have been undertaken to evaluate circRNAs for their applicability as biomarkers and disease modifiers. A growing number of studies have reported the involvement of circRNAs across features of CVD, indicating diagnostic potential as well as potential relevance as regulators of biology (223). Sequencing data revealed more than 15,000 circRNAs present in the human heart, some in high abundance (224). A number of studies have described cardiac circRNAs to be involved in MI-related apoptosis in the myocardium (225, 226) and circRNA MICRA was identified to predict left ventricular dysfunction in MI patients (227). The results were validated in a different study where circRNA MICRA was reported to improve risk stratification of post-MI patients (228). Recently, cardiac circRNAs were assessed for their detectability in the circulation after MI in a controlled stepwise approach (74). Interestingly, none of the screened and validated circRNAs were identified as well-enough detectable in neither plasma nor serum to be used as circulating cell-free biomarkers. The findings question the validity of quantifying circulating circRNAs in cell-free body liquids using currently available technology. In fact, when studying literature regarding circulating circRNAs including the abovementioned studies regarding circRNA MICRA, an interesting fact can be observed: all circulating circRNA biomarker studies report their findings in whole blood samples—containing a large number of circulating cells instead of cell-free serum or plasma. The use of whole blood samples in the assessment of disease biomarkers yields a risk of confounding by cells such as platelets and leukocytes. Thus, the assessment of circRNAs as circulating biomarkers in CVD currently suffers from detectability problems and efforts to improve detectability are needed to further evaluate this issue.

## Discussion

The identification and further exploration of biomolecules suitable as biomarkers for specific disease is a complex process, which requires numerous prerequisites to be met such as detectability in the circulation, reliable quantification methods, pathophysiologic relation to the suspected disease, and many more (Table 2). Proteins have been evaluated as circulating biomarkers for quite a long time and their quantification methods are established. They have also been analyzed for their applicability in heart failure prognosis with promising results already available. Nevertheless, validation of existing results is crucial and has only just begun in this respect. Interestingly, the added value of promising protein biomarkers on top of classic risk factors still remains limited. Therefore, the exploration of alternative biomarkers is a focus of current biomarker research. Alternatives such as genetic risk scores and also ncRNAs have caught scientists' attention for a few years. Genetic biomarkers provide a promising platform to improve mid- and long-term prognostication of HF, in particular with regards to improving individualized approaches of risk evaluation. On the other hand, the current laboratory methodology for their determination is complex, expensive, and time-consuming, limiting their implementation into clinical routine at the current stage. ncRNAs can be stably detected in the circulation and their potential as circulating biomarkers has been recognized. Several ncRNAs have been studied in the context of CVD. miRNAs, lncRNAs, and circRNAs are the most promising ncRNA species being evaluated for their biomarker potential. Several of them are expressed in a cell type- and tissue-specific manner and are involved in distinct physiological and pathological processes, raising hopes for them to evolve as helpful in diagnosis and prognosis of CVD and HF. Currently, application of ncRNAs in clinical settings is hampered by methodological issues such as lack of harmonized quantification methods and suboptimal detectability in the circulation of some transcripts.

**Table 2:** Characteristics of a biomarker.

**Characteristics of a biomarker**	**Protein biomarkers**	**Genetic biomarkers**	**Non-coding RNA biomarkers**
Pathophysiological reliability	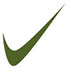	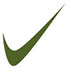	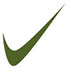
Stability - of biomolecule in body fluids	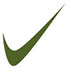	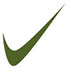	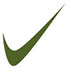
Accessability - through routine clinical procedures	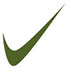	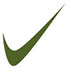	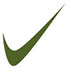
Added value - does the biomarker improve standard clinical evaluation/risk stratification?	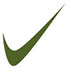	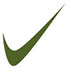	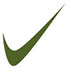
Detectability - is the biomarker stably detectable in target phenotype?	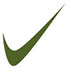	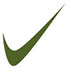	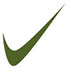
Diagnostic and/or prognostic validation - can the biomarker differentiate affected vs. non-affected individuals?	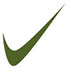	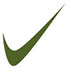	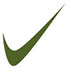
Consensual agreement - are the quantification techniques standardized?	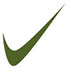	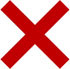	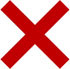
Reference values - are they available and reliable?	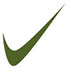	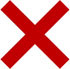	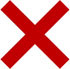
Comparability - of results across centers	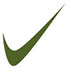	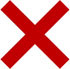	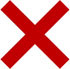
Gender specificity	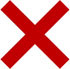	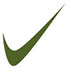	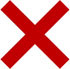
Tested in various ethnicities	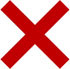	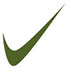	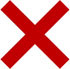

*Protein biomarkers are the most widely used, largely due to methodological advances in their quantification methods and consensual agreement on their detection techniques. Genetic risk scores have the advantage of gender specificity tested across various ethnicities. ncRNAs specifically lack consensual agreement on the quantification methods, reference values and thus comparability of results*.

With respect to HF prognostication, currently the best data is available for NT-proBNP, which has been used in the diagnosis of HF for decades. Its value in HF prognostication has recently been recognized and validation of currently available results seems to be only a matter of time. NT-proBNP has been included into the ESC and AHA guidelines not only because it provides insights into the severity of cardiac damage, but also because current assays allow its detection even at small amounts. But importantly, there is still need for validation of sensitivity and specificity. Large-scale population-based cohort studies applying state-of-the-art laboratory methodology will give the opportunity to identify additional prognostic biomarkers such as genetic biomarkers and validate existing biomarkers for the prognosis of HF. Tissue-specificity seems to play a major role in the application of biomolecules as biomarkers when assessing single markers and it is not surprising that NT-proBNP, as one of only few heart-specific markers, ranks high in the list of biomarker candidates for HF prognostication. On the other hand, given the complex etiology of HF, up until now trials failed to identify single biomarkers in the prognostic assessment of patients with HF. This stretches the importance of the idea to identify complementary biomarkers in order to define biomarker panels as a promising way of improving prognostication of multifactorial disease entities such as HF and argues to include non-organ-specific molecules, which may provide a readout of systemic disease, such as i.e., inflammation, in the seek for biomarkers in HF.

## Author Contributions

AS, TH, and CS wrote the manuscript. TZ and CS edited the manuscript, figures and tables. All authors were responsible for overall editing of the manuscript. All authors contributed to the article and approved the submitted version.

## Conflict of Interest

The authors declare that the research was conducted in the absence of any commercial or financial relationships that could be construed as a potential conflict of interest.
